# 2503. Re-engagement in Care for Patients with Positive Hepatitis C Virus Antibody Test: A Descriptive Study at a Large University Hospital

**DOI:** 10.1093/ofid/ofad500.2121

**Published:** 2023-11-27

**Authors:** Denise Mendoza, Keenan L Ryan, Paulina Deming, Heather Cabos, Kristin Bacon, Bernadette Jakeman

**Affiliations:** University of New Mexico College of Pharmacy, Farmington, NewMexico; University of New Mexico Hospitals, Albuquerque, NewMexico; University of New Mexico, Albuquerque, NewMexico; University of New Mexico College of Pharmacy, Farmington, NewMexico; University of New Mexico College of Pharmacy, Farmington, NewMexico; University of New Mexico, Albuquerque, NewMexico

## Abstract

**Background:**

An HCV micro-elimination project conducted at the University of New Mexico Hospital (UNMH) found a large number of patients with a positive HCV antibody test but no confirmatory HCV RNA test. The purpose of this study was to 1) conduct a telemedicine intervention to re-engage patients with a positive HCV antibody test and no confirmatory HCV RNA test and 2) investigate patient understanding of HCV testing and treatment and identify barriers that impeded patient access to care.

**Methods:**

Through the hospital electronic medical record system, patients with positive HCV antibody tests were identified at UNMH in Albuquerque, NM, USA. Patients were eligible for study inclusion if they were aged > 18 years and had a positive HCV antibody test with no confirmatory HCV RNA documented between January 2019-December 2020. Eligible patients were contacted by phone and asked to participate in a survey on questions addressing socio-demographics, patient awareness of HCV antibody results, knowledge of HCV, and knowledge of current oral HCV treatment options. At survey completion, patients were offered HCV confirmatory testing. Study investigators followed-up with patients who chose to obtain confirmatory testing to provide results and, if appropriate, to refer patients for HCV treatment. Data were analyzed using descriptive statistics to describe the study population. Re-engagement was measured based on the completion of the HCV RNA.

**Methods:**

Inclusion/Exclusion Criteria

**Figure:**
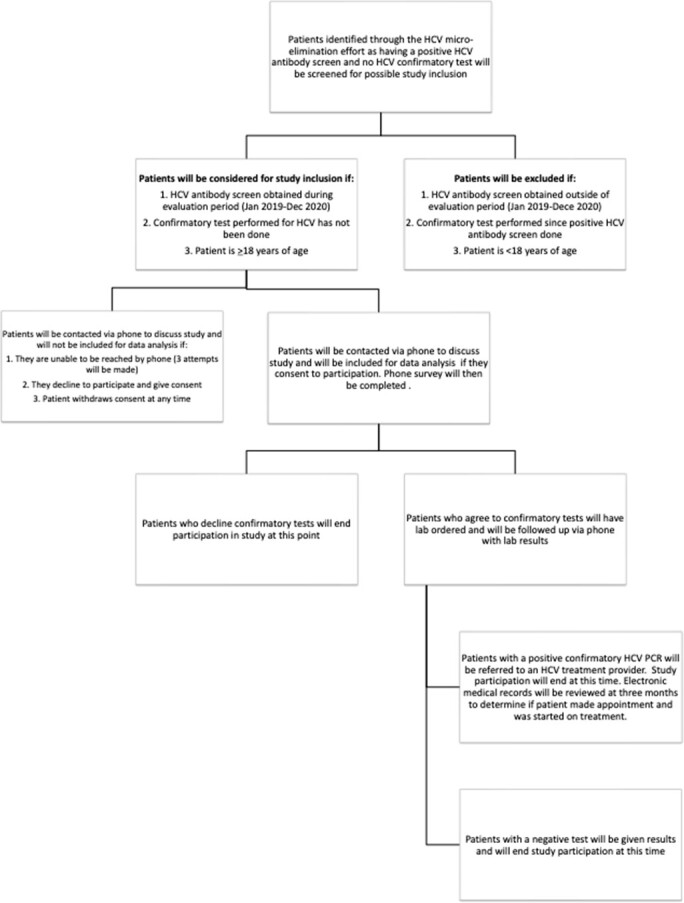

**Results:**

Study investigators attempted to contact 454 patients. From the screened charts, 49% were not reached, 5% passed away, 6% declined, and 4% agreed to participate in the survey. The median age was 51 years. 60% were male and 55% were white. 82% had an annual income of < $35k. All but one of the participants were aware of their HCV antibody test, but many were not aware of chronicity of the virus and available oral treatments. 15 patients had received outside follow up testing and/or treatment. 5 participants were successfully re-engaged in care and received confirmatory testing.

Screening Table

**Figure:**
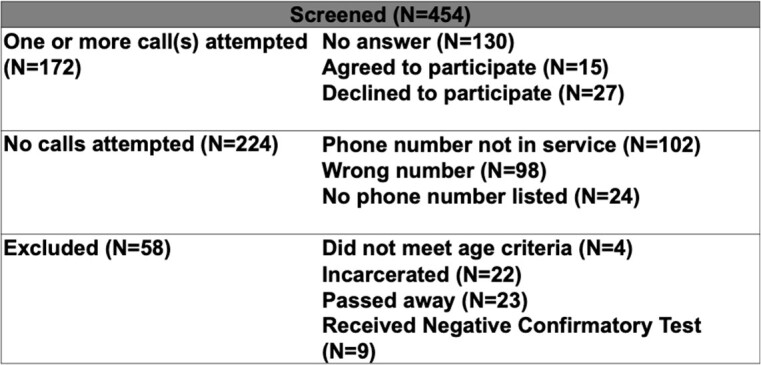

Patient Characteristics

**Figure:**
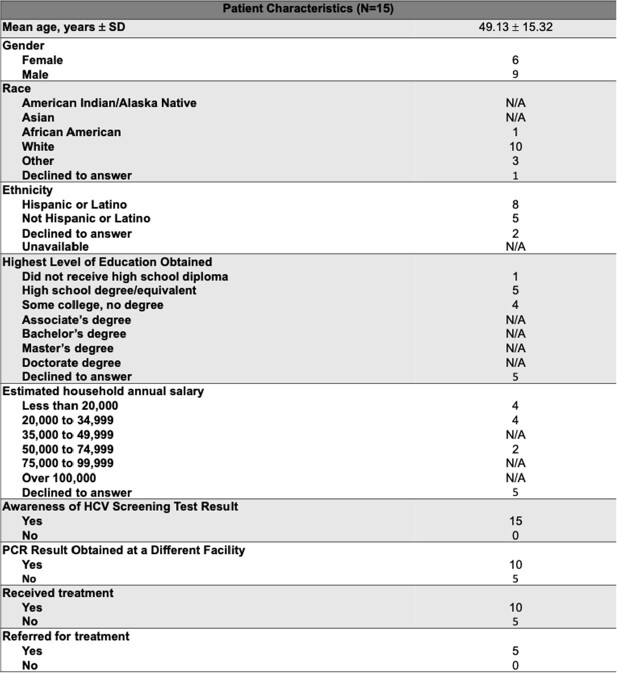

**Conclusion:**

This study highlights the complexity of reaching this patient population to re-engage patients in care for HCV, which is likely due to a number of previously identified socioeconomic factors. This further supports HCV programs that engage patients in care prior to hospital discharge.

**Disclosures:**

**Keenan L. Ryan, PharmD, PhC**, PharmCon: Honoraria **Paulina Deming, PharmD**, Gilead: Advisor/Consultant

